# Multi-GPU Based Parallel Design of the Ant Colony Optimization Algorithm for Endmember Extraction from Hyperspectral Images

**DOI:** 10.3390/s19030598

**Published:** 2019-01-31

**Authors:** Jianwei Gao, Yi Sun, Bing Zhang, Zhengchao Chen, Lianru Gao, Wenjuan Zhang

**Affiliations:** 1 Institute of Remote Sensing and Digital Earth (RADI), Chinese Academy of Sciences (CAS), Beijing 100094, China; gaojw@radi.ac.cn (J.G.); chenzc@radi.ac.cn (Z.C.); gaolr@radi.ac.cn (L.G.); zhangwj@radi.ac.cn (W.Z.); 2 China Academy of Space Technology (CAST), Beijing 100081, China; zoesun99@126.com; 3 University of Chinese Academy of Sciences, Beijing 100049, China; 4 College of Computer Science and Software Engineering, Computer Vision Research Institute, Shenzhen University, Shenzhen 518060, China

**Keywords:** hyperspectral images, endmember extraction, multi-GPU, ant colony optimization (ACO), parallel computing

## Abstract

Spectral unmixing is a vital procedure in hyperspectral remote sensing image exploitation. The linear mixture model has been widely utilized to unmix hyperspectral images by extracting a set of pure spectral signatures, called endmembers in hyperspectral jargon, and estimating their respective fractional abundances in each pixel of the scene. Many algorithms have been proposed to extract endmembers automatically, which is a critical step in the spectral unmixing chain. In recent years, the ant colony optimization (ACO) algorithm has been developed for endmember extraction from hyperspectral data, which was regarded as a combinatorial optimization problem. Although the ACO for endmember extraction (ACOEE) can acquire accurate endmember results, its high computational complexity has limited its application in the hyperspectral data analysis. The GPUs parallel computing technique can be utilized to improve the computational performance of ACOEE, but the architecture of GPUs determines that the ACOEE should be redesigned to take full advantage of computing resources on GPUs. In this paper, a multiple sub-ant-colony-based parallel design of ACOEE was proposed, in which an innovative mechanism of local pheromone for sub-ant-colonies is utilized to enable ACOEE to be preferably executed on the multi-GPU system. The proposed method can avoid much synchronization among different GPUs to affect the computational performance improvement. The experiments on two real hyperspectral datasets demonstrated that the computational performance of ACOEE significantly benefited from the proposed methods.

## 1. Introduction

Hyperspectral sensors can acquire hundreds of contiguous spectral bands for the same area, which provide abundant spectral information about the surface of the Earth. Owing to the narrow band interval adopted in the hyperspectral sensors, it is difficult for sensors to improve their spatial resolution greatly. Therefore, mixed pixels, a mixture of more than one ground object in a single pixel, widely exist in hyperspectral images. The existence of mixed pixels is an intractable problem for hyperspectral data applications [1]. Spectral unmixing is an important technique for hyperspectral remote sensing image analysis [2], which decomposes the spectrum of a mixed pixel into a collection of constituent spectra (i.e., endmembers) and a set of corresponding fractions (i.e., abundances). Endmember extraction is a vital step in spectral unmixing.

A majority of studies of endmember extraction are based on the linear spectral mixture model (LSMM) [2,3]. In LSMM, the spectrum of each pixel can be approximated as a linear addition of endmember spectra according to their fractional abundances in this pixel. As shown in Equation (1), x is one spectrum with *B* bands; *m* is the endmember number; $e_{i}\left(i=1,2,3,\cdots ,m\right)$ stands for the *i*th endmember spectrum of ground objects in the instantaneous field of view (IFOV); $\alpha_{i}\left(i=1,2,3,\cdots ,m\right)$ indicates the abundance of the *i*th endmember in this mixed pixel; ε is the random error.

(1) $$x=\sum_{i=1}^{m}\alpha_{i}e_{i}+\varepsilon .$$

According to Equation (1), two constraints are generally applied in fractional abundance estimation, which are the abundance nonnegativity constraint (ANC) and abundance sum-to-one constraint (ASC), respectively defined in Equations (2) and (3) [4]. Without ASC and ANC, the problem of estimating the abundance can be solved by the least squares (LS) method. If ASC is considered, the solution is called “sum-to-one constrained least squares” (SCLS). It can be called “fully-constrained least squares” (FCLS), if both ASC and ANC are considered.

(2) $$\alpha_{i}\geq 0,\forall i$$

(3) $$\sum_{i=1}^{m}\alpha_{i}=1$$

Many endmember extraction algorithms have been proposed in the past thirty years, e.g., the N-FINDR [5], the pixel purity index (PPI) [6], the vertex component analysis (VCA) [7], the automated morphological endmember extraction (AMEE) [8], the minimum volume simplex analysis (MVSA) [9], and some other new algorithms proposed recently [10,11,12,13]. Swarm intelligence algorithms in artificial intelligence were newly introduced for endmember extraction from hyperspectral images [14,15,16,17,18]. The ant colony optimization algorithm for endmember extraction (ACOEE), a representative endmember extraction method based on swarm intelligence algorithms, utilizes artificial ants to imitate the natural way ants select routes. Owing to the features such as distribution, positive feedback, global search, and stability, ACOEE can extract endmembers accurately and improve extraction precision [14]. However, one issue of ACOEE is its high computational amount, which seriously impacts its application in hyperspectral data analysis.

High-performance computing based on parallel computing architectures, especially commodity graphic processing units (GPUs), is an effective solution to these high computational complexity problems of hyperspectral endmember extraction algorithms [19,20,21,22,23,24,25,26]. Due to the low computational efficiency, many research works have been devoted to the parallel ACO algorithm based on the parallel technology of the multicore CPU, or the single GPU, or the combination of the two [27,28,29,30]. Different from these methods, the most time-consuming task in ACOEE is the calculations of the objective function, e.g., the root-mean-squared error (RMSE) between the remixed image and the original image. B. Zhang et al. [15] introduced parallel computing technology on GPUs to improve the computing performance of ACOEE, and the experiments demonstrated that the processing time of ACOEE was significantly reduced due to the parallel implementation on GPUs. However, the processing time of ACOEE on a single-GPU device (G-ACOEE) was still too high for general application. For the purpose of improving the computing power, multi-GPU systems were used in recent years. In this paper, multi-GPU parallel computing technology was utilized in order to further reduce the processing time of ACOEE, and an innovative parallel implementation based on multiple sub-ant-colonies was designed to make the best use of computing resources in the multi-GPU system. A mechanism of local pheromone was utilized in sub-ant-colonies. The strategy of pheromone updating and stop rules was expressly redesigned in the proposed algorithm. The proposed method can avoid synchronization among different GPUs to consume too much processing time. Compared with G-ACOEE, the proposed parallel algorithm not only employs the data-level parallelism, but also the task-level parallelism. The experiments demonstrated that the processing time of ACOEE was significantly reduced thanks to the proposed multi-GPU-based parallel design, and moreover, there is great potential to further enhance the searching capability of ACOEE without too much time cost.

The remainder of the paper is organized as follows. The ACOEE algorithm is briefly reviewed in Section 2. Section 3 describes an innovative multiple sub-ant-colony-based parallel design of ACOEE in a multi-GPU system. Section 4 provides an experimental analysis of the proposed method using two well-known hyperspectral datasets. Finally, Section 5 concludes with some remarks and presents several directions for future research.

## 2. Ant Colony Optimization Algorithm for Endmember Extraction

The ant colony optimization (ACO) algorithm is a representative swarm intelligence algorithm [31]. Ants in nature utilize a kind of chemical substance named pheromones for communication. Pheromones are released on the routes passed by moving ants when they search for food. An isolated ant can detect the pheromones on the routes and then randomly select a route based on the pheromone concentrations on the routes. Furthermore, the higher the pheromone concentration is, the more attractive the route is. As time goes on, pheromones will concentrate on the route traveled by the majority of ants; thus, this route will be chosen by more and more ants. This means that a positive feedback system is formed. On the other hand, the natural dissipation of the pheromones produces a similar, but opposite effect. If a route is less selected by ants, pheromones on this route will disappear gradually, and thus, fewer ants will select those paths. The shortest route between the ants’ nest and the food source can be found through this positive feedback system.

The ACO algorithm solves optimization problems by analysis and simulation of ant colony behavior. In an ACO algorithm, optimization problems are modeled just as natural ants searching for the shortest route between their nest and the food source, and artificial ants are utilized to imitate the procedure of natural ants selecting routes. In the environment artificial ants are living in, routes and time are both discrete. This means that a route consists of several discrete sections, and the artificial ant can select one section in a time unit. Several time units are needed for searching a route. Owing to the features such as positive feedback, distribution, and global search, the ACO algorithm for endmember extraction (ACOEE) from hyperspectral remote sensing images was proposed [14] and improved in the literature [15]. The improved ACOEE optimized the heuristic information to improve the algorithm accuracy and utilized an elitist strategy and GPUs to reduce the processing time. ACOEE also showed the capacity to find the optimal possible combination out of a set of candidate endmembers generated by other methods automatically, as well as robustness in dealing with noise and outliers [32].

With the assumption of the existence of pure pixels, extracting the endmembers’ set can be looked upon as a combinational optimization problem. In ACOEE, endmember extraction was transformed into the optimization problem by building the feasible solution space and defining the objective function. A directed graph denoted by *G* was constructed first to generate the solution space. Each vertex in *G* corresponds to a pixel in the hyperspectral image. All vertexes in *G* are connected. Artificial ants can release pheromones on the edges between vertexes. Assume *m* endmembers are to be extracted, and then, the route constructed by an artificial ant should contain *m* different vertexes in *G*. An artificial ant randomly selects vertexes according to the concentration of pheromones on edges. Each edge in *G* is initialized with the same concentration of pheromones at the preliminary stage. When the ant arrives at vertex $v_{i}$ after t−1(t≥1) times of moving, the transition probability of moving from $v_{i}$ to the next vertex $v_{j}$ is defined as:

(4) $$p_{ij}\left(t\right)=\frac{\tau_{ij}\left(t\right)}{\sum_{j\in allowed_{t}}\tau_{ij}\left(t\right)},\forall j\in allowed_{t}.$$

In Equation (4), $allowed_{t}$ indicates the set of vertexes that can be reached by this ant from $v_{i}$ at time *t*, i.e., all the pixels that have not been reached by the ant, and $\tau_{ij}\left(t\right)$ is the concentration of pheromones in the edge $<v_{i},v_{j}>$. Once all ants accomplish their tasks searching for the shortest routes, the routes will be evaluated according to the objective function. Then, pheromones on edges are updated as:

(5) $$\tau_{ij}\left(t+1\right)=\rho \tau_{ij}\left(t\right)+\Delta \tau_{ij}\left(t\right),$$

where ρ is the pheromones’ dissipation factor and $\Delta \tau_{ij}\left(t\right)$ is the pheromones released on the edge $<v_{i},v_{j}>$ at time *t*. Let $f_{t}$ and $route_{t}$ respectively denote the optimal objective function value of all selected routes and the corresponding route. $\Delta \tau_{ij}\left(t\right)$ is defined as:

(6) $$\Delta \tau_{ij}\left(t\right)=\left\{\begin{array}{cc} Q/f_{t} & \;\mathrm{if}\;<v_{i},v_{j}>\in route_{t}\\ 0 & \;\mathrm{if}\;<v_{i},v_{j}>\notin route_{t} \end{array}\right.,$$

where *Q* is a constant that controls the range of pheromone adjustments $\Delta \tau_{ij}\left(t\right)$. According to a route selected by one artificial ant (i.e., *m* endmembers), a remixed image can be constructed by abundance estimation, and the root-mean-squared error (RMSE) between the remixed image and the original image is adopted as the objective function to evaluate the endmembers’ set. RMSE can be calculated as Equation (7), in which $x_{i}$ and ${\hat{x}}_{i}$ respectively indicate a pixel spectrum vector and its corresponding remixed vector. RMSE can evaluate the quality of selected solutions without any ground truth information. The smaller the RMSE is, the better the endmember set is. It has been demonstrated that RMSE was an effective evaluating indicator for endmember extraction [14,15,33]. Equation (6) can guarantee that the lower the objective function value is, the more released pheromones there are.

(7) $$RMSE=\frac{1}{n}\sum_{i=1}^{n}{∥x_{i}-{\hat{x}}_{i}∥}_{2}$$

Up to now, an iteration has been completed, and the ants begin another iteration aiming at the optimal solution. The algorithm stops when the iteration number reaches a predetermined maximum number (10,000 in the practical experiments), or the same solution is obtained in several consecutive iterations (e.g., four).

## 3. Multi-GPUs-Based Parallel Design of ACOEE

As a result of the increasing demand for high-performance graphic computing and deep learning computing, the computing power of graphic processing units (GPUs) has made great achievements and been widely applied for general purposes in recent years. Considering the excellent features, e.g., light weight, small size, and low cost, GPUs are widely utilized to improve the computing performance in hyperspectral data applications [19,20,21,22,23,24,34,35,36,37,38,39]. Compute unified device architecture (CUDA) (http://docs.nvidia.com/cuda/cuda-c-programming-guide/index.html), introduced by NVidia corporation, provides a development environment for creating high performance GPU-accelerated applications.

**Figure 1:**
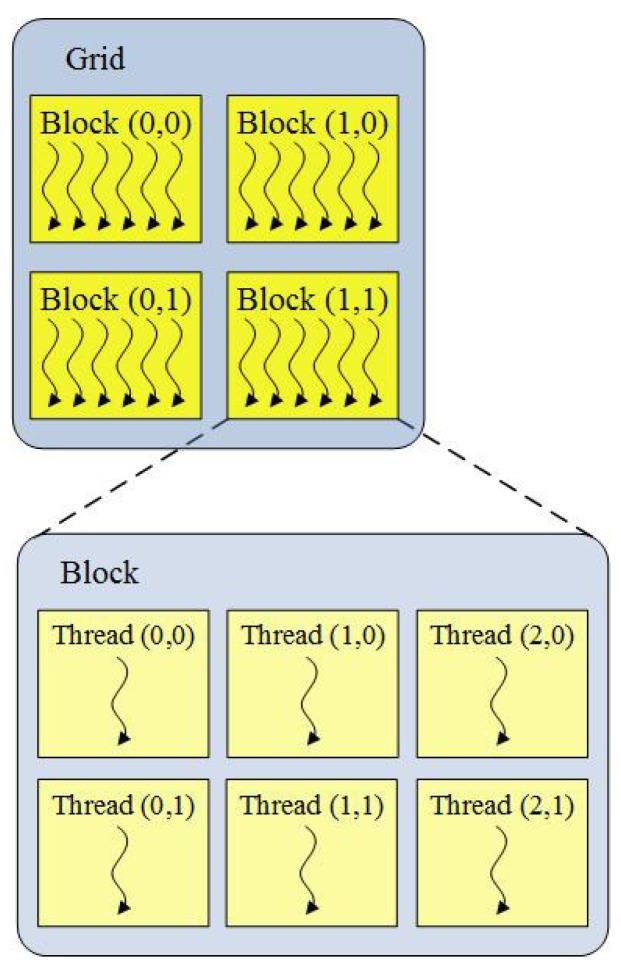
Illustration of a grid of blocks of threads.

Because major transistors are devoted to data processing, GPUs are not good at controlling flow. Therefore, GPUs are generally used as a coprocessor with powerful computing capabilities in a computer system. In the context of CUDA, the tasks with a high computational amount are executed on GPUs in parallel (device), while the task of controlling algorithm flow is accomplished on the CPU (host). Due to thousands of stream processors on one GPU, GPU is capable of executing massively light-weight threads of the same program. A program executed in the GPU is called a kernel, which is executed in parallel using up to thousands of threads. In other words, multiple threads execute the same instructions, but operate on different elements of data. Considering the characteristics of GPUs, CUDA arrange threads in a form of a grid of blocks (see Figure 1).

A global memory, a shared memory, and registers constitute the memory architecture of GPU. The global memory, connected via a high-bandwidth bus to the chip of GPU, can be accessed by all threads; registers and the shared memory are the limited on-chip memory resource, which can be accessed only by a certain thread or threads in a thread block. Furthermore, threads in a block are distributed on the same stream multiprocessor and thus must share the limited registers and shared memory. The thread structure of a kernel should be designed on the basis of the computing resource of GPUs and the characteristics of algorithms.

Considering the complexity of the ACOEE, the improved ACOEE [15] maintained the basic structure of the original ACOEE (O-ACOEE), while most computing tasks of each ant were completed on the device in parallel. Aiming at taking full advantage of computing resources on the GPU, the computing tasks completed in parallel were sufficiently optimized. The processing time per iteration in ACOEE was significantly reduced as a result of the GPU-based parallel technique. The multi-GPU parallel technique is used to further improve the computing performance of ACOEE. In a computing system with multiple GPUs, the computing power increases several times compared with the system with a single GPU. However, the hardware architecture becomes more complex in a multi-GPU system, and parallel algorithm running in this system must avoid too frequent synchronization among the GPUs. Therefore, the parallel implementation of ACOEE is redesigned correspondingly in order to get an excellent computational performance.

### 3.1. Parallel Design Based on Multiple Sub-Ant-Colonies

**Figure 2:**
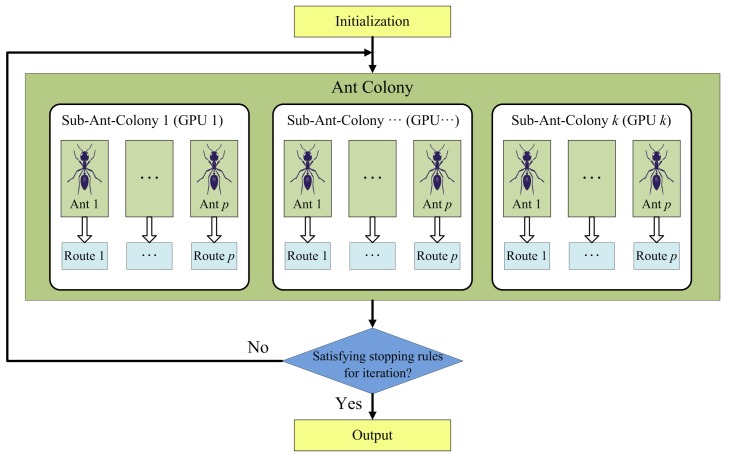
Schematic overview of the parallel design based on multiple sub-ant-colonies.

The multi-GPU system provides much more computing resource than the single-GPU system, and therefore, the computing task allocation of ACOEE must be redesigned. If the tasks with a high computational amount of each ant are allocated to different GPUs, just as in the parallel implementation of ACOEE in a single-GPU system, a number of synchronous operations will happen among GPUs and the host. In synchronous operations, data transmission via PCIe bus consumes much time. Furthermore, the computational amount allocated to each GPU is so small that GPUs cannot be fully utilized. For this reason, it is not a recommended choice. In this subsection, a parallel design based on multiple sub-ant-colonies is proposed (see Figure 2).

In the proposed design, the algorithm flow is still controlled on the host, but an ant colony is equally divided into several sub-ant-colonies, searching for routes in the same directed graph. The computing tasks of sub-ant-colonies are respectively allocated to different GPUs, which means that the computing tasks of ants in a sub-ant-colony are accomplished in the same GPU. Therefore, the computing resource of each GPU is efficiently used, and the number of synchronous operations is significantly reduced. As a result, the strategy of pheromone updating and stop rules of the algorithm should be redesigned to adapt to the multiple sub-ant-colonies strategy.

1. The strategy of pheromone updating:

Pheromone, utilized in communications among ants, is a key factor of the positive feedback mechanism in ACOEE. Once ants complete searching for a route in an iteration, the amount of pheromones will be updated, and then, ants can search for better routes based on the latest pheromones on the graph in the next iteration.

The computing tasks of sub-ant-colonies are carried out on different GPUs, so the pheromone information is stored in the corresponding global memory of different GPUs. Once the pheromone data are updated on one GPU, the other sub-ant-colonies should synchronize the pheromone data on the corresponding GPUs. In a synchronization, the pheromone data are transferred from each GPU to the host via PCIe bus and then transferred back to each GPU after being updated in the host. Synchronization is a time-consuming operation. For this reason, it is not an economical choice to synchronize the pheromone information in each iteration. As a result, an innovative strategy of pheromone updating is proposed to deal with this problem.

(1) There are two kinds of pheromones in the graph, i.e., global pheromone and local pheromone. The global pheromone is visible for all ants and can be updated by all ants. Each ant can utilize and update the local pheromone in a sub-ant-colony. The global pheromone and local pheromone are both initialized with the same value.
(2) Ants search for routes according to the local pheromone. At the end of each iteration, the local pheromone is updated on the basis of Equation (5) and (6). $f_{t}$ and $route_{t}$ in Equation (6) respectively indicate the optimal objective function value of all selected routes searched in this sub-ant-colony and its corresponding route.
(3) After *SyncNum* iterations, the local pheromone data of all sub-ant-colonies are copied to the host. The global pheromone data are updated to the mean value of the local pheromone data of different sub-ant-colonies. Then, the latest global pheromone data are copied back to each device to update the local pheromone data of all sub-ant-colonies.
(4) If the stopping condition is not satisfied, the host will control the devices to execute the next *SyncNum* iterations.

In this strategy of pheromone updating, ants search for routes on the basis of the local pheromone in each sub-ant-colony, and the pheromone data are synchronized among different sub-ant-colonies at the regular time. This not only guarantees that the ants can utilize the global optimal pheromone information for searching for routes in a timely manner, but also avoids too much synchronization increasing the time consumption.

2. Stop rules of the algorithm:

In O-ACOEE, the algorithm stops when the same best solution in several consecutive iterations is obtained or the iterations number increases to the maximum number. In the proposed parallel implementation of ACOEE on the multi-GPU system, the ants exchange information with ones in the same sub-ant-colony in most iterations and can exchange information among different sub-ant-colonies every *SyncNum* iterations. For convenience, *SyncNum* iterations are also called a synchronous cycle. After a synchronous cycle, the global best solution is updated by comparing the latest local best solutions searched by all the ants. Therefore, it is designed such that the stop conditions are determined once the global best solution is updated in each synchronous cycle instead of each iteration in the proposed algorithm. If the same best solution in several consecutive synchronous cycles or the iterations number increases to a preset maximum number, the algorithm will stop, and this solution is recognized as the final global optimal solution, i.e., endmembers. The pseudocode for the proposed stop rules of MG-ACOEE is shown in Pseudocode 1.
  Pseudocode 1:  Step 0: Initializing parameters, allocating device memory, and transferring data to devices in the host. The parameters: *IterationMax* (the preset iteration maximum number), *ConverMax* (the convergence synchronous cycle number), and *SyncNum* are initialized. *TotalIterNum* = 0; *ContiConverTimes* = 0.  Step 1: For each sub-ant-colony, the local pheromone data are synchronized with the global pheromone data. *IterNum* = 0.  Step 2: For each sub-ant-colony, feasible solutions are obtained by ants. The local best solution is updated through comparing the RMSE values of feasible solutions, and then local pheromone data are updated. *IterNum = IterNum+1*. *TotalIterNum=TotalIterNum+1*.  Step 3: For each sub-ant-colony, if *IterNum* > *SyncNum*, copy the local best solution and local pheromone data to the host, and then, go to Step 4; else, go to Step 2.  Step 4: For the host, the best solution in this synchronous cycle is obtained through comparing the local best solutions. If this solution is the same as the global best solution, *ContiConverTimes=ContiConverTimes* + 1; else *ContiConverTimes* = 0, and update the global best solution.  Step 5: For the host, if *ContiConverTimes* = *ConverMax* or *TotalIterNum* = *IterationMax*, the algorithm stops, and the best solution in the last synchronous cycle is recognized as the final global optimal solution; else, update the global pheromone data, and go to Step 1.

These stop rules are adapted to the multiple sub-ant-colonies-based parallel implementation, and the time of determining the stopping conditions is substantially reduced in the proposed method.

### 3.2. Parallel Implementation of ACOEE on the Multi-GPU System

**Figure 3:**
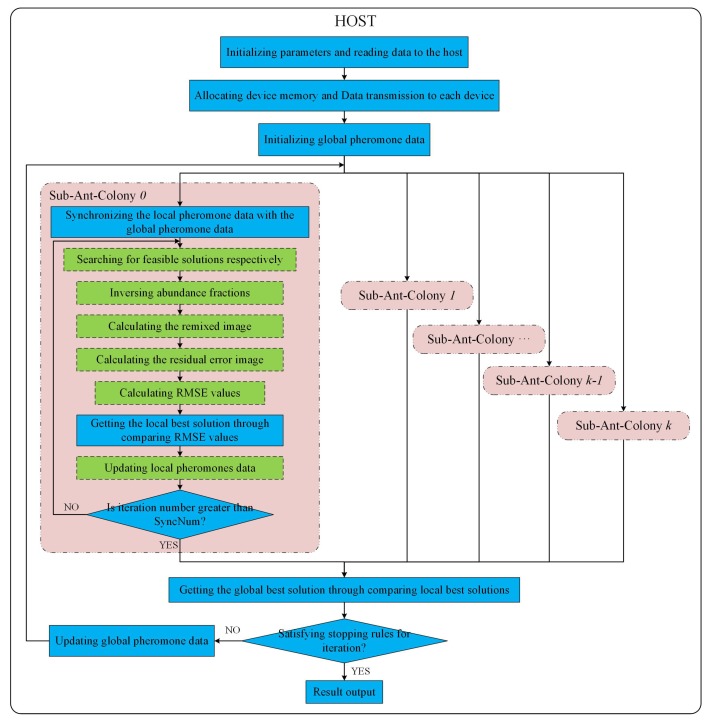
Schematic overview of the implementation of multi-GPU ACO for endmember extraction (MG-ACOEE).

According to the design in Section 3.1, the multi-GPU technique is utilized to implement ACOEE in a parallel way, aiming at reducing the processing time per iteration in ACOEE. The parallel implementation of ACOEE on a multi-GPU system is called MG-ACOEE, while the version on a single-GPU system is named as G-ACOEE. In MG-ACOEE, the host is in charge of flow controlling, parameter initialization, data transfer, and determining stop conditions. For these sub-ant-colonies, OpenMP threads are utilized to control the task flows, and all the time-consuming tasks are implemented in the GPUs. Figure 3 shows the schematic overview of the implementation of MG-ACOEE. In Figure 3, the operations in the blue solid boxes and in the green dashed boxes are respectively executed in the host and devices (i.e., GPUs).

1. Initialization:

Firstly, the major parameters should be initialized, such as endmembers’ number, the number of sub-ant-colonies (GPUs), *SyncNum*, and so on. The hyperspectral image is read from the hard disk to the host memory in the following step. Then, the space on the devices’ global memory is allocated, and data are copied from the host to the devices. Compared to O-ACOEE, the device memory allocation and data transmission in MG-ACOEE consume extra overhead time. Therefore, most computing tasks are completed on the device, and thus, the addresses of device memories are passed among different kernel functions, avoiding data transmission between the host and the devices. Since the global pheromone data are visible for all ants, they are allocated and initialized in the host. The local pheromone data are initialized with the same preset constant in each GPU device by kernel *UpdatePro*, in which the thread number is equal to the number of candidate endmembers.

2. Iterative process of each sub-ant-colony:

As shown in Figure 3, an OpenMP thread is utilized to control the task flow of a sub-ant-colony and to launch kernels on a GPU to accomplish the time-consuming tasks of this sub-ant-colony. For example, the task flow of Sub-ant-colony 0 is controlled in the OpenMP Thread 0, in which the major computing tasks of Sub-ant-colony 0 are allocated to the GPU 0.

In this thread, the global pheromone data are firstly copied to the device to update the local pheromone data. Then, artificial ants in the sub-ant-colony search for routes (i.e., solutions or endmembers) via a kernel function executed on a GPU. In this kernel function, each ant is assigned to a block of threads to search for a route in parallel. In practice, there were *m* thread blocks in this kernel, and the threads’ number in each block was set to the candidate endmember number. The cuRAND library (https://developer.nvidia.com/curand) was used to generate the random floating point numbers.

Once the endmembers are selected, the following step is to calculate the objective functions of the selected endmembers. The RMSE values’ calculation of endmembers is the most time-consuming step in MG-ACOEE. In order to calculate RMSE values, the abundance fractions of pixels in the hyperspectral image should be calculated to begin with. A 1-element is added to each endmember vector due to the sum-to-one constraint in abundance inversion. A kernel function is executed to implement this procedure in parallel, in which the block number and the thread number in a block are respectively equal to the endmember number and the bands number in the hyperspectral image. This means that a block of threads is utilized for adding a 1-element to each endmember vector. Two abundance constraints are considered in this paper. If the new endmember vectors are adopted in the unconstrained least squares method to obtain the abundance fractions, the abundances obey the ASC. In this procedure, the matrix inversion is implemented by a proposed kernel function on a GPU, while the product of two matrices is calculated by the function *cublasSgemm* from the highly efficient cuBLAS library (https://developer.nvidia.com/cublas). If the new endmember vectors are adopted in the methods proposed in Ref. [4], the abundances obey the ASC and ANC. The kernel *CUDAAbundanceNNLS* is executed on a GPU to accomplish the least squares method considering the nonnegativity constraint, in which a thread is utilized to calculate the abundance vector of a pixel. There are 256 threads in a block, and the block number is correspondingly set to (*MixedPixelNum* + 255)/256.

The following steps are to calculate the remixed image and the residual error image, which are also implemented by functions *cublasSgemm* and *cublasSaxpy* from the cuBLAS library. A kernel function named *CUDARmseFromResidualErrorMatrix* is executed on the GPU to calculate the RMSE values from the residual error image. In this kernel, the thread number in a block is set to 512, and the block number is correspondingly set to (*MixedPixelNum* + 511)/512.

The best solution in the current iteration is selected by comparing the RMSE values after all RMSE values of endmembers are obtained. The local pheromone data are then updated on the GPU in parallel according to the best solution in the current iteration. If the iteration number is no greater than *SyncNum*, the next iteration starts.

3. Synchronization among different sub-ant-colonies:

After a synchronous cycle, a synchronization among different sub-ant-colonies is carried out, and then, the stop conditions of the algorithm will be checked. Once all different sub-ant-colonies have completed a synchronous cycle, the local best solutions and local pheromone data of different sub-ant-colonies are transferred from devices to the host. Then, the global best solution is obtained through comparing the local best solutions in the host. In the following step, the stop conditions will be determined. If the stop conditions are not satisfied, the mean value of local pheromone data of different sub-ant-colonies will be utilized to update the global pheromone data, and then, a new synchronous cycle for all sub-ant-colonies continues. If the stop conditions are satisfied, pixels in the latest global best solution are considered as endmembers.

## 4. Experiments and Discussion

In this section, experiments on real hyperspectral data are presented to evaluate the endmember extraction accuracy and parallel performance of the efficient implementation of ACOEE.

### 4.1. Computing Facilities and Dataset

The computer utilized in the experiments was equipped with two Intel Xeon E5-2620 CPUs, 128 GB RAM, and eight NVIDIA TITAN Xp GPUs. Table 1 shows the features of the GPUs utilized in the experiments, which were connected to the computer using the PCI-Express 2.0 bus. The experiments were performed on the Ubuntu 16.04 operating system, in which the CUDA development environment 8.0 was installed. The proposed MG-ACOEE algorithm was carried out using CUDA C language, while O-ACOEE and G-ACOEE were carried out separately using MATLAB and CUDA C for comparison purposes.

**Figure 4:**
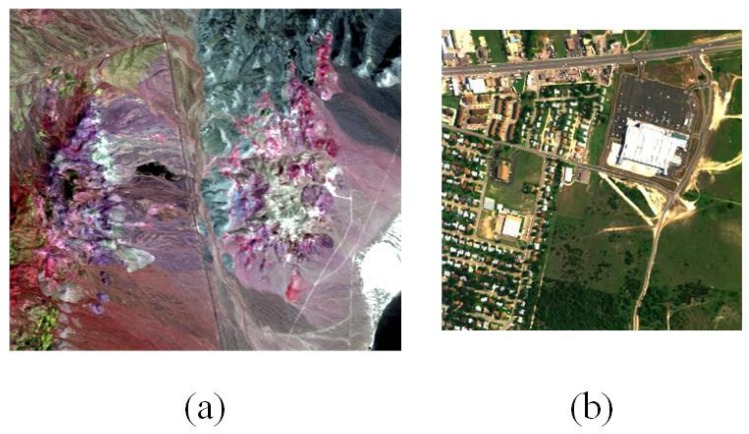
(**a**) False color composition of the Cuprite dataset. (**b**) True color composition of the Urban dataset.

Two popular hyperspectral datasets were adopted in the experiments for evaluating the performance of the proposed algorithm (see Figure 4). The first one was the well-known Cuprite scene acquired by the Airborne Visible/Infrared Imaging Spectrometer (AVIRIS) [40]. There were several minerals in this district, such as alunite, calcite, kaolinite, and muscovite. The scene utilized in this section comprised 400 × 350 pixels, and 50 bands ranging from 1.99–2.48 μm. The other one was the Urban data captured by Hyperspectral Digital Imagery Collection Experiment (HYDICE) in October 1995, which is one of the most widely-used hyperspectral datasets for hyperspectral unmixing research [41,42,43]. The image is of size 307×307, and there were 210 bands ranging from 400–2500 nm in each pixel. Due to dense water absorption and atmospheric effects, the bands 1–4, 76, 87, 101–111, 136–153, and 198–210 were removed, and 162 bands remained in these data. The main ground objects in this scene include asphalt road, grass, tree, and roof.

Aiming at reducing the size of feasible solution space, 80 pixels with the highest pixel purity index were extracted as candidate endmembers by the pixel purity index (PPI) algorithm [6]. The number of endmembers in the Cuprite dataset was first estimated by HySime [44], which provided an estimation of m=8 endmembers. According to the literature [41,42], there are two versions of the ground truth of Urban data, which contain four and six endmembers, respectively. The number of endmembers in the Urban data was set to six in this paper. In order to reduce the computational complexity, 2000 pixels were uniformly sampled from the original hyperspectral datasets, instead of all pixels, for fully-constrained abundance inversions in ACOEE. The times in all the tables of this section are in seconds.

### 4.2. Endmember Extraction Accuracy and Parallel Computing Performance

The endmember extraction accuracy and parallel performance of O-ACOEE, G-ACOEE, and MG-ACOEE are compared in this section. The spectral angle distances and RMSE were adopted to evaluate endmember accuracy. Because ACOEE is a random search algorithm, the number of iterations varied with each run. Therefore, the computing performance was evaluated by the time per iteration (TPI). The mean and standard variances of the metrics over five runs for these algorithms were given in the experiments.

The ant number in these algorithms was set to 256. The number of sub-ant-colonies in MG-ACOEE, i.e., the number of GPUs, was eight. This means that the ant number in each sub-ant-colony of MG-ACOEE was 32. In MG-ACOEE, the number of iterations in a synchronous cycle, e.g., *SyncNum*, was set to four.

Four highly representative minerals (i.e., alunite, calcite, kaolinite, and muscovite) in the cuprite mining district were utilized for endmember accuracy comparison. Table 2 and Table 3 respectively report the spectral angle distances between USGS mineral spectra and their corresponding endmembers extracted by O-ACOEE, G-ACOEE, and MG-ACOEE with only ASC and full constraint. Asphalt road, grass, tree, and roof in the Urban data were considered for endmember extraction accuracy comparisons between the extracted endmembers and the ground truths, which are shown in Table 4 and Table 5. From the analyses of spectral angle distances and RMSE values in these tables, it could be found that the three algorithms all successfully extracted the considered endmembers in the experiments on Cuprite data and Urban data, and MG-ACOEE obtained comparable results, compared with O-ACOEE and G-ACOEE.

However, the computing performance of MG-ACOEE was superior to that of O-ACOEE and G-ACOEE in the experimental results. The total time, iteration numbers, and TPI of these three algorithms are reported in Table 6, Table 7, Table 8 and Table 9. It should be noted that the total time not only included the processing time on the host and the device, but also the time of data allocations and data transmissions. In these experiments, both the total time and TPI were greatly reduced in MG-ACOEE. Thanks to the multi-GPU features, TPIs of MG-ACOEE with only ASC or full constraint were respectively reduced 6.80- and 7.40-times for Cuprite data and 7.38- and 6.87-times for Urban data, when compared with G-ACOEE.

In summary, the parallel computing performance of MG-ACOEE was significantly improved under the premise of ensuring endmember accuracy.

### 4.3. Influence of Key Parameters

Key parameters in MG-ACOEE can affect the iteration number, TPI, and even the searching ability. In this subsection, more experiments of MG-ACOEE with only the ASC constraint were carried out in order to evaluate the influence of the number of GPUs (*GPUsNum*), the number of ants in a sub-ant-colony (*AntsNum*), and the number of iterations in a synchronous cycle (*SyncNum*).

The mean and standard variances of the RMSE, iteration number, total time, and TPI over five runs for these algorithms are given in the following experiments. For convenience, iteration number and total time were abbreviated to IN and TT.

1. Influence of *GPUsNum*:

In MG-ACOEE, the ant colony can be divided into more sub-ant-colonies as *GPUsNum* increases, if there is a certain number of ants in an ant colony. Four experiments were executed to evaluate the influence of the number of GPUs (*GPUsNum*), in which the number of ants in the ant colony was 256, and the *GPUsNum* was respectively set to 1, 2, 4, and 8. Obviously, it was G-ACOEE when the *GPUsNum* was equal to one. *AntsNum* was respectively 128, 64, and 32, when *GPUsNum* was 2, 4, and 8. *SyncNum* was set to four in these experiments.

Table 10 and Table 11 show the RMSE, IN, TT, and TPI of these experiments separately on Cuprite and Urban data. From these tables, we can observe that the total time and times per iteration significantly decreased as *GPUsNum* increased, while the RMSE was basically stable. This means that the computing performance of ACOEE can benefit from the proposed parallel strategy, in which multiple GPUs were utilized to accomplish the computing tasks of the subsidiary ant colonies in an ant colony.

**Figure 5:**
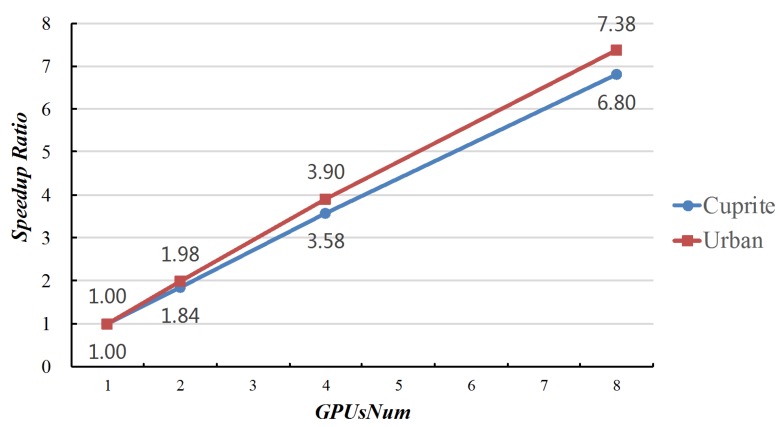
Speedup ratios of MG-ACOEE with different *GPUsNum* and G-ACOEE.

Figure 5 reveals the speed ratios of MG-ACOEE with different *GPUsNum* and G-ACOEE. When the *GPUsNum* increased, the speed ratio correspondingly increased. However, the speed ratios could not come up to *GPUsNum*, due to the time cost of data synchronization among different GPUs. It also can be found that the speed ratios in the experiments of Urban data were higher than the ones in the experiments of Cuprite data. That is because there were more bands in the Urban data, and more time was spent on searching for the optimal solution instead of data synchronization.

2. Influence of *AntsNum*:

If the the number of sub-ant-colonies (*GPUsNum*) was fixed, the number of ants per sub-ant-colony (*AntsNum*) not only determines the total number of the ant colony, but could also impact the parallel performance of MG-ACOEE. In this subsection, *GPUsNum* was set to eight, and five experiments were set up to evaluate the influence of *AntsNum*, in which the *AntsNum* was respectively set to 4, 12, 20, 28, and 32. The ant numbers in the colony correspondingly were 32, 96, 160, 224, and 256. *SyncNum* was set to four in these experiments.

**Figure 6:**
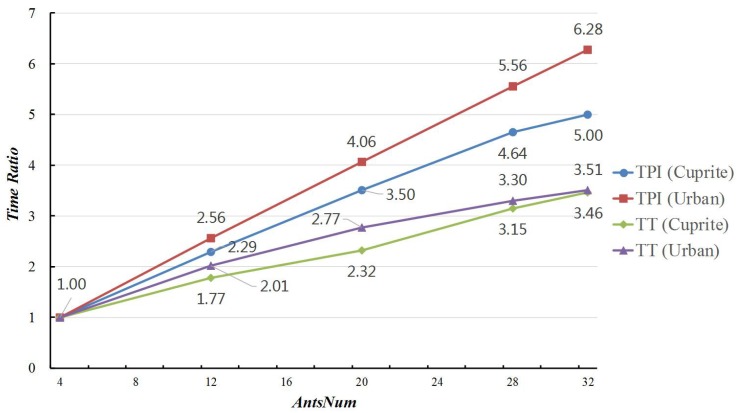
Time ratios of MG-ACOEE with different *AntsNum* and G-ACOEE.

Table 12 and Table 13 report the average values and standard variances of RMSE, IN, TT, and TPI in these experiments. As *AntsNum* increased from 4–32, the RSME value decreased from 3.052–3.018 in the experiments on Cuprite data, which meant that the searching ability of MG-ACOEE distinctly enhanced. Since there were more ants in the colony, the computing amount searching for optimal solution accordingly increased. If the TT and TPI of MG-ACOEE with *AntsNum* = 4 was taken as a benchmark, Figure 6 reveals the time ratios of MG-ACOEE with different *AntsNum* and the benchmark. In this figure, it can be found that the TPI time ratios for Cuprite and Urban data, indicated by the *TPI(Cuprite)* and *TPI(Urban)* curves, were less than the *AntsNum* ratios in the MG-ACOEE with different *AntsNum*. For example, if *AntsNum* = 32, the *AntsNum* ratios was eight, while the TPI time ratios for Cuprite and Urban data were respectively 5.00 and 6.28. That was because the computing time searching for the optimal solution increased as *AntsNum* increased, while the synchronizing time cost essentially was unchanged. This also means that the percentage of synchronizing time in TT decreased accordingly. For the same reason, the TPI time ratios on Cuprite data were less than the ones on Urban data. As a result of enhanced searching ability in an iteration, less iterations were needed in MG-ACOEE with more ants in the colony to obtain the optimal solution. Therefore, TT time ratios were even less than TPI time ratios for both Cuprite and Urban data, shown by the *TT(Cuprite)* and *TT(Urban)* curves.

3. Influence of *SyncNum*:

In MG-ACOEE, ants in a sub-ant-colony complete *SyncNum* iterations in a synchronous cycle, and then, the global best solution and global pheromone data are updated and the convergence conditions checked. *SyncNum* represents the frequency of synchronizing operations in MG-ACOEE, which are closely related to time per iteration and the convergence rate of the algorithm.

In this subsection, six experiments were carried out to evaluate the influence of *SyncNum*. *GPUsNum* and *AntsNum* were separately set to eight and 32 in all the experiments, and *SyncNum* was respectively set to 4, 8, 16, 32, 64, and 96. In the same way, the algorithms were run five times separately on Cuprite and Urban data, and then, the average values and standard variances of RMSE, IN, TT, and TPI are reported in the Table 14 and Table 15.

**Figure 7:**
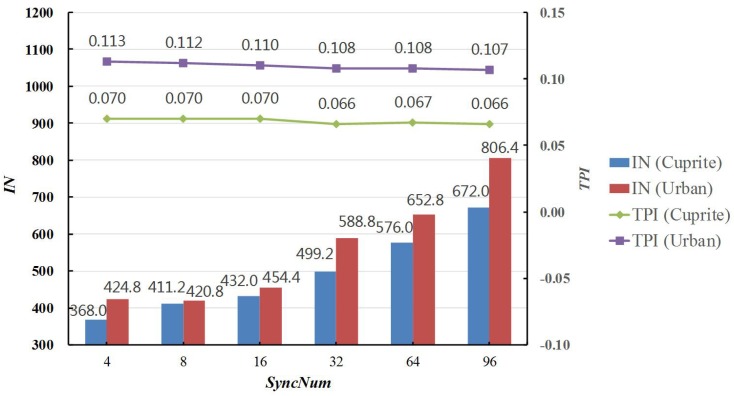
Time Ratios of MG-ACOEE with different *SyncNum* and G-ACOEE.

The variations of TPI and IN in Cuprite and Urban experimental results also can be found in Figure 7. It was revealed that TPI slightly decreased, whereas IN considerably increased, when *SyncNum* increased from 4–96. This was because the synchronizing number and correlative time cost was reduced accordingly. However, it should be particularly noted that there were fewer chances to check the convergence conditions of the algorithm as *SyncNum* went up. Therefore, the convergence conditions were more difficult to satisfy, and IN notably increased by more than 80%; as a result, more time was spent on obtaining the optimal solutions. Thanks to the more full searching in the solution space, the RMSE values were slightly reduced in both the Cuprite and Urban experimental results.

### 4.4. Discussions

In the experiments on two real hyperspectral datasets, both endmember extraction accuracy and parallel performance were evaluated, and then, the influences of key parameters, i.e., *GPUsNum*, *AntsNum*, and *SyncNum*, were analyzed. For a colony with a fixed number of ants, the computing performance of MG-ACOEE was significantly improved owing to the multi-GPU parallel computing technology, when compared with O-ACOEE and G-ACOEE. Moreover, the advantage of MG-ACOEE on computing performance would be greater if more GPUs were utilized, while maintaining their endmember extraction accuracy. Therefore, it is proposed that *GPUsNum*, i.e., the number of sub-ant-colonies, should be set as big as possible in a multi-GPU computing system. If *GPUsNum* was fixed, total times and times per iteration did not increase linearly as *AntsNum* increased, because less time was utilized for data synchronization. It was a cost-efficient choice to set *AntsNum* = 32 in the MG-ACOEE for the reason that the RMSE of endmembers could be reduced owing to more ants used for searching for the optimal solution. In the experiments, *SyncNum* increasing could make the times per iteration slightly decrease and the iteration number considerably increase. Considering the lower RMSE values along with fuller searching in the solution space, *SyncNum* is recommended to be set to 32.

From the experimental analyses, it could be found that the RMSE values were closely related to the ant number in the colony and the iteration number. In other words, fuller searching in the solution space was conducive to obtaining higher precision endmember results. MG-ACOEE has shown high computational efficiency and has great potential to further improve the searching capability without too much time cost.

## 5. Conclusions

This paper proposes a multi-GPU-based parallel design to the ACOEE algorithm aiming at improving its computing performance. In order to take full advantage of the computing resource in a multi-GPU system, a parallel design based on multiple sub-ant-colonies was applied, in which the computing tasks of multiple sub-ant-colonies were allocated to different GPUs. The strategy of pheromone updating and stop rules of the algorithm was redesigned to adapt to the sub-ant-colonies. The experiments on the well-known Cuprite and Urban data demonstrated that the computational performance of ACOEE was significantly improved owing to the proposed methods, and preferable endmember extraction accuracy and parallel performance can be obtained via setting proper key parameters in MG-ACOEE.

Due to the tremendous advantage in computing performance, MG-ACOEE has the potential to improve the endmember extraction accuracy ulteriorly, which means that more GPUs and more ants will be considered to improve the searching ability of MG-ACOEE in future work. We also will focus on applying the multi-GPU parallel technology to accelerate the efficiency of other information extraction algorithms on hyperspectral remote sensing data.

## Figures and Tables

**Table 1:** Main GPU features.

	NVIDIA TITAN Xp
CUDA core	3840
Boost Clock (MHz)	1582
Memory Amount (GB)	12
Memory Speed (Gbps)	11.4
Memory Interface width (bit)	384
Memory Bandwidth (GB/S)	547.7

**Table 2:** Comparison of original (O)-ACOEE, G-ACOEE, and MG-ACOEE (abundance sum-to-one constraint (ASC)) on endmember extraction accuracy for Cuprite data.

Algorithms	Spectral Angle Distance (×10^−2^)					RMSE
	Alunite GDS84	Calcite WS272	Kaolinite KGa-1	Muscovite GDS107	Mean	
O-ACOEE	9.20 ± 1.95	10.03 ± 0.34	9.40 ± 0.77	9.82 ± 1.15	9.61 ± 1.05	3.030 ± 0.016
G-ACOEE	7.97 ± 1.74	10.13 ± 0.26	9.05 ± 0.95	9.56 ± 0.69	9.18 ± 0.91	3.024 ± 0.012
MG-ACOEE	7.19 ± 0	9.89 ± 0.31	9.79 ± 0.11	9.56 ± 0.69	9.11 ± 0.28	3.018 ± 0.007

**Table 3:** Comparison of O-ACOEE, G-ACOEE, and MG-ACOEE (full constraint) on endmember extraction accuracy for Cuprite data.

Algorithms	Spectral Angle Distance (×10^−2^)					RMSE
	Alunite GDS84	Calcite WS272	Kaolinite KGa-1	Muscovite GDS107	Mean	
O-ACOEE	7.27 ± 0.18	10.12 ± 0.25	9.92 ± 0.13	10.13 ± 0.60	9.36 ± 0.29	3.300 ± 0.020
G-ACOEE	7.42 ± 0.41	10.35 ± 0.25	9.53 ± 0.52	10.45 ± 0.12	9.43 ± 0.33	3.295 ± 0.020
MG-ACOEE	7.19 ± 0	10.36 ± 0.30	9.71 ± 0.48	10.46 ± 0.13	9.43 ± 0.23	3.295 ± 0.021

**Table 4:** Comparison of O-ACOEE, G-ACOEE, and MG-ACOEE (ASC) on endmember extraction accuracy for Urban data.

Algorithms	Spectral Angle Distance (×10^−2^)					RMSE
	Asphalt Road	Grass	Tree	Roof	Mean	
O-ACOEE	19.10 ± 0	8.70 ± 0	6.92 ± 1.73	5.61 ± 0	10.08 ± 0.43	8.155 ± 0.018
G-ACOEE	19.10 ± 0	8.70 ± 0	7.54 ± 2.79	5.61 ± 0	10.20 ± 0.70	8.156 ± 0.018
MG-ACOEE	19.10 ± 0	8.70 ± 0	8.19 ± 1.41	5.61 ± 0	10.40 ± 0.35	8.142 ± 0.014

**Table 5:** Comparison of O-ACOEE, G-ACOEE, and MG-ACOEE (full constraint) on endmember extraction accuracy for Urban data.

Algorithms	Spectral Angle Distance (×10^−2^)					RMSE
	Asphalt Road	Grass	Tree	Roof	Mean	
O-ACOEE	18.83 ± 0	8.70 ± 0	5.66 ± 0	23.70 ± 0	14.22 ± 0	10.173 ± 0.652
G-ACOEE	18.83 ± 0	8.70 ± 0	5.66 ± 0	23.70 ± 0	14.22 ± 0	10.168 ± 0.007
MG-ACOEE	18.83 ± 0	8.70 ± 0	5.66 ± 0	23.70 ± 0	14.22 ± 0	10.165 ± 0.007

**Table 6:** Comparison of O-ACOEE, G-ACOEE, and MG-ACOEE (ASC) on computing performance for Cuprite data.

Algorithms	Iteration Numbers	Total Time	TPI (Time per Iteration)
O-ACOEE	284.0 ± 35.49	9259.83 ± 1468.86	32.605 ± 1.140
G-ACOEE	324.6 ± 50.07	154.64 ± 24.12	0.476 ± 0.011
MG-ACOEE	424.8 ± 25.35	29.87 ± 1.68	0.070 ± 0.001

**Table 7:** Comparison of O-ACOEE, G-ACOEE, and MG-ACOEE (full constraint) on computing performance for Cuprite data.

Algorithms	Iteration Numbers	Total Time	TPI (Time per Iteration)
O-ACOEE	299.0 ± 28.45	67,868.65 ± 9388.48	226.500 ± 14.280
G-ACOEE	370.0 ± 53.58	1991.81 ± 304.16	5.379 ± 0.059
MG-ACOEE	444.8 ± 49.10	323.73 ± 40.58	0.727 ± 0.012

**Table 8:** Comparison of O-ACOEE, G-ACOEE, and MG-ACOEE (ASC) on computing performance for Urban data.

Algorithms	Iteration Numbers	Total Time	TPI (Time per Iteration)
O-ACOEE	264.6 ± 49.35	18,155.67 ± 3244.98	68.616 ± 0.898
G-ACOEE	271.0 ± 35.43	226.07 ± 27.56	0.834 ± 0.008
MG-ACOEE	368.0 ± 22.05	41.58 ± 2.41	0.113 ± 0.001

**Table 9:** Comparison of O-ACOEE, G-ACOEE, and MG-ACOEE (full constraint) on computing performance for Urban data.

Algorithms	Iteration Numbers	Total Time	TPI (Time per Iteration)
O-ACOEE	219.0 ± 17.26	44,685.95 ± 4504.05	204.142 ± 13.994
G-ACOEE	226.4 ± 20.40	1221.10 ± 115.46	5.392 ± 0.034
MG-ACOEE	285.6 ± 30.93	225.89 ± 54.34	0.785 ± 0.127

**Table 10:** Comparison of different *GPUsNum* in MG-ACOEE on the parallel performance for Cuprite data. IN, iteration number; IT, total time.

GPUsNum	1	2	4	8
**RMSE**	3.024 ± 0.012	3.026 ± 0.009	3.022 ± 0.009	3.018 ± 0.007
**IN**	324.6 ± 50.07	316.8 ± 30.51	368.8 ± 48.69	424.8 ± 25.35
**TT**	154.64 ± 24.13	82.02 ± 7.37	49.10 ± 5.59	29.87 ± 1.68
**TPI**	0.476 ± 0.011	0.259 ± 0.011	0.133 ± 0.004	0.070 ± 0.001

**Table 11:** Comparison of different *GPUsNum* in MG-ACOEE on the parallel performance for Urban data.

GPUsNum	1	2	4	8
**RMSE**	8.170 ± 0.021	8.149 ± 0.018	8.149 ± 0.017	8.142 ± 0.014
**IN**	271.0 ± 35.43	284.8 ± 11.43	312.8 ± 20.61	368.0 ± 22.05
**TT**	226.07 ± 27.56	119.85 ± 5.43	66.92 ± 4.29	41.58 ± 2.41
**TPI**	0.834 ± 0.008	0.421 ± 0.003	0.214 ± 0.001	0.113 ± 0.001

**Table 12:** Comparison of different *AntsNum* in MG-ACOEE on the parallel performance for Cuprite data.

AntsNum	4	12	20	28	32
**RMSE**	3.052 ± 0.016	3.035 ± 0.025	3.025 ± 0.008	3.021 ± 0.007	3.018 ± 0.007
**IN**	594.4 ± 57.28	477.6 ± 75.92	411.2 ± 52.24	414.4 ± 61.43	424.8 ± 25.35
**TT**	8.62 ± 0.48	15.29 ± 2.28	25.33 ± 3.49	27.13 ± 4.33	29.87 ± 1.68
**TPI**	0.014 ± 0.001	0.032 ± 0.002	0.056 ± 0.003	0.065 ± 0.002	0.070 ± 0.001

**Table 13:** Comparison of different *AntsNum* in MG-ACOEE on the parallel performance for Urban data.

AntsNum	4	12	20	28	32
**RMSE**	8.255 ± 0.117	8.163 ± 0.014	8.156 ± 0.017	8.149 ± 0.018	8.142 ± 0.014
**IN**	665.6 ± 51.64	524.0 ± 57.24	452.0 ± 11.03	390.4 ± 37.57	368.0 ± 22.05
**TT**	11.86 ± 0.96	23.85 ± 2.30	32.88 ± 0.74	39.14 ± 3.60	41.58 ± 2.41
**TPI**	0.018 ± 0	0.046 ± 0.001	0.073 ± 0	0.100 ± 0.001	0.113 ± 0.001

**Table 14:** Comparison of different *SyncNum* in MG-ACOEE on the parallel performance for Cuprite data.

SyncNum	4	8	16	32	64	96
**RMSE**	3.018 ± 0.007	3.020 ± 0.009	3.015 ± 0.008	3.012 ± 0.004	3.012 ± 0.002	3.012 ± 0.004
**IN**	424.8 ± 25.35	420.8 ± 53.69	454.4 ± 21.70	588.8 ± 15.68	652.8 ± 74.64	806.4 ± 97.9
**TT**	29.87 ± 1.68	29.61 ± 3.48	31.77 ± 1.81	38.68 ± 1.02	43.69 ± 4.99	52.84 ± 6.21
**TPI**	0.070 ± 0.001	0.070 ± 0.001	0.070 ± 0.002	0.066 ± 0	0.067 ± 0.001	0.066 ± 0

**Table 15:** Comparison of different *SyncNum* in MG-ACOEE on the parallel performance for Urban data.

SyncNum	4	8	16	32	64	96
**RMSE**	8.142 ± 0.014	8.142 ± 0.014	8.142 ± 0.014	8.141 ± 0.013	8.135 ± 0	8.135 ± 0
**IN**	368.0 ± 22.05	411.2 ± 33.79	432.0 ± 44.11	499.2 ± 47.89	576.0 ± 40.48	672.0 ± 60.72
**TT**	41.58 ± 2.41	45.86 ± 3.45	47.39 ± 4.80	54.16 ± 4.84	62.01 ± 4.53	71.95 ± 6.27
**TPI**	0.113 ± 0.001	0.112 ± 0.001	0.110 ± 0.001	0.108 ± 0.001	0.108 ± 0.001	0.107 ± 0.001
